# Single-Stapled Double Purse-String Anastomotic (SIA) Technique in Robotic Malignant Sigmoid Resections—A Danish Single-Center Study

**DOI:** 10.3390/jcm15031100

**Published:** 2026-01-30

**Authors:** Helene Juul Würtz, Flemming Hansen Dall

**Affiliations:** 1 Department of Surgery, Vejle Hospital, Lillebaelt University Hospital of Southern Denmark, 7100 Vejle, Denmark; flemming.hansen.dall@rsyd.dk; 2 Department of Regional Health Research, Faculty of Health Sciences, University of Southern Denmark, 5000 Odense, Denmark; 3 Danish Colorectal Cancer Center South, Vejle Hospital, Lillebaelt University Hospital of Southern Denmark, 7100 Vejle, Denmark

**Keywords:** sigmoid cancer, single-stapling anastomosis, purse-string suture, robotic anastomotic technique

## Abstract

**Background:** Stapled end-to-end anastomosis has a leakage rate close to 10%. Studies indicate that most leaks occur where stapler lines overlap. The single-stapled double purse-string suture technique (SIA) eliminates stapler line overlaps in low anterior resection (LAR) and may thereby decrease leakage rates. **Methods:** This single-arm, single-center study prospectively and consecutively registered all patients with sigmoid colonic cancer planned for robotic sigmoid resection with primary anastomosis over a two-year period. The primary outcome was time to perform SIA and secondary outcomes were total operative time and short-term complications. **Results:** The study group consisted of twenty-one patients. The median time to perform SIA was 11.5 min. Two patients (9.5%) experienced 30-day postoperative complications. One patient had ischemic bowels and pneumonia postoperatively and another had an anastomotic leakage grade C. **Conclusions:** This study has several limitations, including a small sample size, lack of comparator group, and short follow-up period. However, these preliminary results may indicate the SIA technique to be feasible without prolonging the operation substantially. Larger series are, however, evidently needed to evaluate the SIA technique in further detail to elucidate whether the technique is generalizable and safe.

## 1. Introduction

Colon cancer surgery has developed greatly over the recent decades. Usually, bowel continuity is restored by the construction of an anastomosis. Originally, bowel anastomoses were hand-sewn, but for many years now, an anastomosis can also be performed using a stapler, which can seal and transect the bowel at the same time. There are various options as to the orientation of the anastomosis (end-to-end, end-to-side, side-to-side) and whether it is constructed outside the abdomen (extracorporeal) or within the abdominal cavity (intracorporeal) during minimally invasive surgery.

Stapled end-to-end anastomosis (EEA) in low anterior resection (LAR) of the rectum became possible after the introduction of the circular stapler in 1979. After resection of the rectum, the continuity of the intestine could be restored using an EEA stapler and a purse-string suture in both the proximal colon and the rectal stump. This was, in effect, the introduction of the SIA technique in conventional open surgery [1]. A randomized prospective evaluation comparing the EEA stapling technique versus a hand-sewn anastomosis showed equivalence in safety and postoperative complications [2].

A decade later, the linear stapler was introduced, making it easier to seal and transect the distal rectum. The combination of the stapled distal rectum and the use of an EEA stapler resulted in the double-stapling technique [3], which is widely used today. Unfortunately, the anastomotic leakage rates in an anterior rectal resection remain at almost 10% despite many efforts to prevent this severe complication [4,5,6]. Ikeda et al. made an interesting discovery when investigating clinical colorectal anastomotic leakages endoscopically. All the leakages occurred in the circular stapler line and half of all the leakages were located where stapler lines overlapped, suggesting that these points could be the weakest part of the anastomosis [7]. In 2017, Lee et al. compared anastomotic leakage rates between overlapping stapler lines in 128 consecutive left colon and rectal cancer patients, who underwent laparoscopic rectal resection using the double-stapling technique [8]. The study demonstrated significantly fewer anastomotic leakages in the group with no overlapping stapler lines [8]. Several series have recently described the single-stapled double purse-string anastomosis in the low colorectal anastomoses after low rectal resections, showing it to be reproducible and safe in selected patients [9]; also, a smaller cohort describing a robotic intracorporeal single-stapling anastomosis in left-sided colorectal cancers has been reported to be viable and safe [10].

To our knowledge, no previous trials have investigated single-stapled double purse-string technique in sigmoid resections for malignant tumors. Eliminating overlapping stapler lines may reduce leak rates in sigmoid resections. Therefore, the objective of our study was to develop and test the feasibility of the SIA technique in robotic surgery for malignant tumors of the sigmoid colon before conducting a larger interventional study.

## 2. Materials and Methods

### 2.1. Design

This single-arm, single-center study prospectively registered all consecutive patients with a sigmoid colonic cancer planned for robotic sigmoid resection with primary anastomosis over a two-year period. The primary outcome was time to perform SIA and secondary outcomes were operative time, blood loss and short-term complications. Patients were followed for 30 days postoperatively.

The study protocol was approved by the Regional Committees on Health Research Ethics for Southern Denmark (IRB approval number: 19/30237) and by the Danish Data Protection Agency, and rules and regulations regarding data management were followed. The study was registered at Clinicaltrials.gov (NCT04223141) prior to patient inclusion. All patients gave written informed consent prior to the operation.

### 2.2. Patients and Setting

#### Study Group

Inclusion criteria were patients over 18 years of age, who were able to give informed consent. All patients had an endoscopically and biopsy-verified sigmoid cancer and curative treatment with minimally invasive surgery using end-to-end anastomosis that was deemed possible by a multidisciplinary team. Two surgeons experienced in robotic colorectal surgery performed all anastomoses. They trained and calibrated the technique by performing the first 10 anastomosis in unison and then alternating which surgeon performed the anastomosis. This was done to minimize performance bias.

Between March 2020 and July 2022, thirty-nine patients were included in the study. The patients received an enema in the morning prior to surgery. Postoperatively, the patients received magnesium hydroxide 1 g twice a day and were discharged once they had bowel movement, a sufficient oral intake and were mobilized. The first five patients were allocated to pilot testing, as planned, to align and test the study setup. The setup and anastomotic techniques were then standardized and used consecutively on the study population.

### 2.3. Surgical Technique

#### 2.3.1. Conventional Robotic Technique

The patient was positioned in the Trendelenburg position with their left side slightly up, and the robotic ports were placed diagonally in a line from the right iliac fossa to just over the umbilical level on the left side. Once the peritoneum was medially incised, the mesorectal plane was identified and dissected to the mid rectum. The ureter was identified. The inferior mesenteric artery and vein were sealed with the vessel sealer distal to the left colic artery. The mesocolic dissection plane was followed, mobilizing the sigmoid colon cranially, allowing the sigmoid enough length to achieve an oncologically correct resection and length of the colon to make the anastomosis. The splenic angle was released only if tension across the anastomosis was assessed. The mesorectum was transected with vessel sealer and the rectum was transected with a Sureform stapler. A Pfannenstiel incision was performed and draped with wound protector, and the colon was extracted and transected. The stapler anvil was secured in the proximal colon with a purse-string suture with monofilament suture. The colon was then repositioned inside the abdomen and the bowel ends were joined together by a stapled circular anastomosis.

#### 2.3.2. The SIA Technique

**Figure 1:**
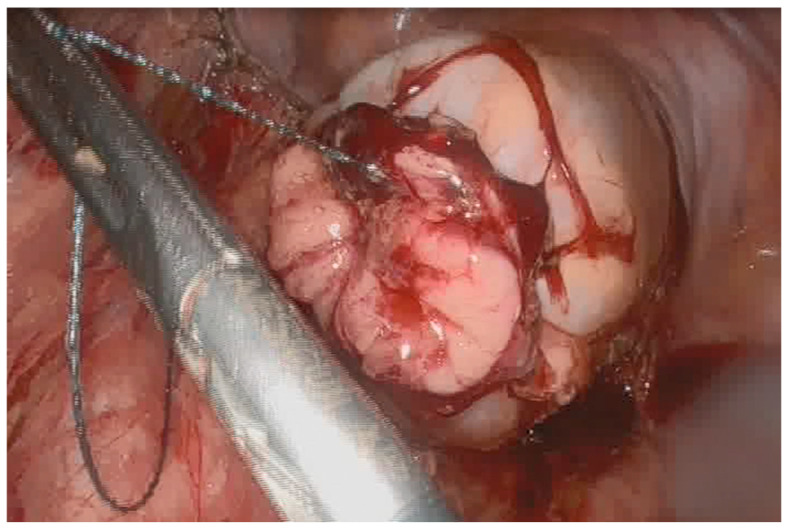
Tightening of the purse-string suture of the distal rectal end of the anastomosis during SIA technique.

**Figure 2:**
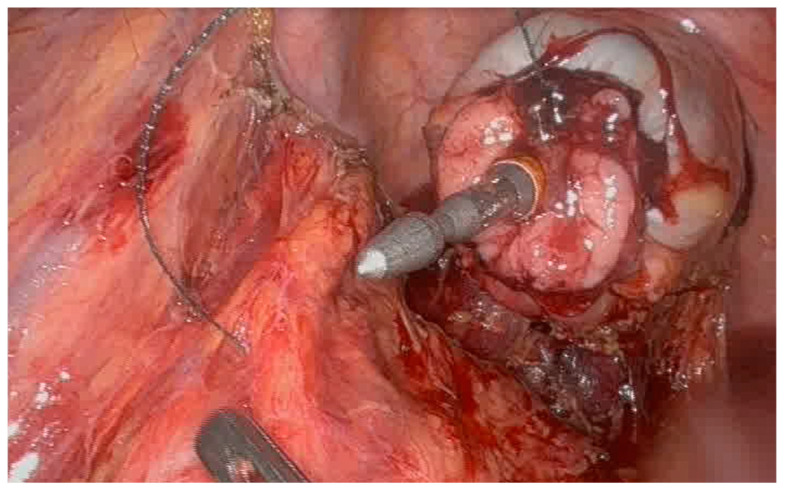
Connector brought forth centrally in the tightened purse-string suture of the distal rectal end of the anastomosis during SIA procedure.

The rectum was transected with a Sureform stapler as mentioned above and subsequently cleaned with an aqueous chlorhexidine solution to diminish possible contamination. Butyl scopolamine was administered to reduce muscular contractions while performing the SIA procedure. The linear stapler line was resected with robotic scissors and a purse-string suture with a v-loc 3-0 suture was placed in the rectum about 5 mm from the edge of the open end with the robotic needle-driver. Starting at the most ventral point, a running suture with 5–8 serosal stitches was placed in the circumference before locking the v-loc and tightening the suture. The suture was ultimately passed through the center of the tightened suture and only the needle was removed, leaving a long suture end to help guide the circular stapler through the rectal stump and bring the connector forth centrally in the tightened suture (Figure 1 and Figure 2). The specimen was removed transabdominally, as described above in the conventional robotic technique. The stapler device and the anvil were connected, resulting in a single-stapled circular anastomosis. As in the conventional method, a leak test was performed to ensure the integrity of the anastomosis. The leak test was made by introducing a catheter and insufflating air, and simultaneously having the anastomosis submerged in water to evaluate for bubbles indicating leakage. Endoscopy was not routinely performed.

The patient received 3 g cefuroxime and 1.5 g metronidazole intravenously preoperatively. Postoperatively, the patient was given oral magnesium oxide 1 g twice a day and oral paracetamol 1 g four times a day. If additional pain medication was required, morphine 5–10 mg was the first-line medicine.

## 3. Results

Thirteen patients were excluded perioperatively from the study due to the tumor not being located in the sigmoid as expected, conversion to open operation, the presence of peritoneal carcinomatosis, and one failure of the double purse-string where it was not repeated but conventional double-stapling technique was performed. The purse-string suture did not close the bowel completely when pulled tight. The traditional technique was performed to ensure patient safety as performing a double-stapled anastomosis would involve another more distal stapling on the rectal stump. In hindsight, it may have been possible to redo the purse-string suture with closer stitches; however, we applied a safety-first principle in this study of performing this innovative technique. The procedure completion rate for SIA technique was nevertheless 26 out of 27, resulting in a technical success rate of >95%.

As the first five patients were allocated to pilot testing as described above, the final study group with completed SIA consisted of 21 patients. Their characteristics and peri- and postoperative data are shown in Table 1. The study cohort had a median age of 70 years, 52% were male, 67% were ASA 2, 86% had Performance Status (PS) 0 and median BMI was 24.8. Sixty-two percent of the patients had tumor stage T3 or above.

The median time to perform SIA was 11.5 min (range 7–45 min). One value was missing and therefore excluded. The median operative time was 190 min and median blood loss was 20 mL. The median length of stay was 3 days. Two patients (9.5%) experienced complications within 30 days. One patient developed ischemia of the afferent colon and pneumonia postoperatively. The ischemia of the colon was possibly due to local damage of the vascularization of the colon perioperatively. Another patient had a grade C anastomotic leakage. Both patients had reintervention, resulting in colostomy in both cases.

## 4. Discussion

To our knowledge, this is the first documented series describing the short-term outcomes of single-stapled double purse-string anastomotic technique in sigmoid resections.

Fewer wound infections, shortened length of stay, and less pain are some of the advantages gained from changing from open surgery to minimally invasive surgery [11]. Laparoscopic colectomy was introduced in the 1990s and is proven to be as safe and effective as open surgery [12]. However, laparoscopy has its limitations in small spaces, such as the pelvic space, where transection of the rectum and anastomosis can be difficult steps as angulation of instruments and visualization can be a problem. Robot-assisted minimally invasive surgery overcomes these limitations and makes the laparoscopic SIA technique easier to perform. This is partly due to the dexterity that comes with the rotation of the wrists in the console and the mobility of the robotic instruments, which is particularly valuable close to the target and in small spaces such as the pelvic space in a male patient. The high-definition three-dimensional surgical visual field and the surgeon’s control of all four robotic arms, including the camera, assists accuracy and speed when performing SIA.

The laparoscopic single-stapled double purse-string suture technique has been described in low anterior resections for rectal cancers. In 2010, this was first reported with three cases reported by Prasad et al. using a robotic technique [13], and in a larger study in a case–control study with 180 patients by Kim et al. in 2013 [14]. In that study [14], 60 patients who received laparoscopic or robotic single-stapled anastomosis were compared with 120 patients who received laparoscopic or robotic double-stapled anastomosis. There was no statistical difference in the postoperative morbidity, including anastomotic leakages, in the length of stay, in the time to first gas passage postoperatively.

In 2017, 82 patients undergoing laparoscopic low anterior resection of sigmoid and rectum with single-stapled anastomosis were compared with 106 patients undergoing double-stapled anastomosis. The distal purse-string suture was performed laparoscopically, and the single-stapled double purse-string technique took, on average, 10 min longer to perform compared to the double-stapling technique. There were no statistically significant differences in overall morbidity including anastomotic leakage rates [15]. The results in our study had similar time to perform SIA technique (median duration of 11.5 min).

The SIA procedure differs from other single-stapled anastomotic procedures described in the literature after total mesorectal excisions in two ways. Firstly, this anastomosis is performed after a sigmoid resection, and therefore, the rectal stump is longer than it would be after a total mesorectal excision. Secondly, the purse-string suture of the distal rectum is placed and tightened without the need of a second clinician introducing the stapler transanally at the time of the purse-string suture placement, saving the resource of a second clinician who would otherwise be occupied holding the stapler transanally as well as the unnecessary potential for lesions to occur from having to keep the stapler in place for approximately 10 min. The suture end remaining in place at the distal rectal stump is then used to guide the stapler when introduced transanally and centralized.

The greatest limitation of our study is the very small sample size of only 21 patients, which only tells us that the technique was feasible in these patients in this setting. It cannot tell us whether it is safe or generalizable to other settings. Another limitation is that there is no direct comparator group to test whether this technique is superior or equivalent to the double-stapling technique. Furthermore, the short-term follow-up period is also a limitation in our study as long-term sequelae such as anastomotic strictures or abnormal bowel function cannot be addressed. Based on our results, we propose a study to include a larger sample size with a double-stapling technique comparator group, with both short- and long-term follow-up.

There are several possible advantages of using the SIA procedure. From an economic perspective, the robotic SIA technique may theoretically result in less use of staplers if shown to be equivalent or superior to the conventional double-stapling technique, thereby reducing costs [16]. However, this would require other methods to close the rectal stump temporarily when transected. Another possible advantage may be avoiding a larger abdominal incision for specimen extraction as the SIA procedure makes it possible to remove the specimen through the rectum.

Finally, in theory, removing overlapping stapler lines eliminates the weakest part of the standard double-stapled anastomosis and may thereby result in a mechanically stronger anastomosis [12], which could subsequently result in fewer anastomotic leakages as described above. Together with the aforementioned advantages using the robotic technique, we therefore believe this may be a feasible alternative technique for sigmoid resection.

## 5. Conclusions

The SIA procedure is reproducible, simple, and does not appear to prolong the operation compared with the conventional double-stapling technique by more than 11.5 min on average. The results indicate that the SIA technique is feasible. However, this is a study of a small sample size. Larger studies with a comparator group and longer follow-up are needed to evaluate whether the SIA technique is generally safe to perform.

## Figures and Tables

**Table 1:** Patient characteristics and outcomes in 21 consecutive patients with sigmoid cancer undergoing robotic sigmoid resection with single-stapled anastomosis.

Patient Characteristics (n = 21)	Distribution	Range
Age (in years)	70 (Median)	35–84
Male sex	11/21 (52.4%)	
BMI	24.8 (Median)	19.6–34.8
Clinical tumor stage		
T1	4/21 (19%)	
T2	4/21 (19%)	
T3	10/21 (48%)	
T4	3/21 (14%)	
ASA score		
1	4/21 (19%)	
2	14/21 (67%)	
3	3/21 (14%)	
4	0	
PS		
0	18/21 (86%)	
1	3/21 (14%)	
2	0	
Operative time (minutes)	190 (Median)	125–303
Time to perform SIA (min)	11.5 (Median)	7–45
Blood loss (ml)	20 (Median)	0–100
LOS (days)	3 (Median)	2–16

BMI = Body Mass Index; ASA = American Society of Anesthesiologists; PS = performance status; LOS = length of stay.

## Data Availability

The data that support the findings of this study are available on request from the corresponding author.

## Abbreviations

The following abbreviations are used in this manuscript:

LAR	Low anterior resection
SIA	Single-stapled double purse-string anastomotic technique
EEA	End-to-end anastomosis
BMI	Body Mass Index
ASA	American Society of Anesthesiologists
PS	Performance status
LOS	Length of stay
